# Editorial for the Special Issue: Molecular Epidemiology, Diagnostics and Management of Respiratory Virus Infections

**DOI:** 10.3390/microorganisms8122041

**Published:** 2020-12-20

**Authors:** Hirokazu Kimura, Yuriko Hayashi, Mitsuru Sada, Haruyuki Ishii, Takeshi Tsugawa, Akihide Ryo

**Affiliations:** 1 Department of Health Science, Graduate School, Gunma Paz University, 1-7-1 Tonyamachi, Takasaki-shi, Gunma 370-0006, Japan; hayashi@paz.ac.jp (Y.H.); rainbow_orchestra716@yahoo.co.jp (M.S.); 2 Department of Microbiology, School of Medicine, Yokohama City University, 3-9 Fukuura, Kanazawa-ku, Yokohama-shi, Kanagawa 236-0004, Japan; aryo@yokohama-cu.ac.jp; 3 Department of Respiratory Medicine, School of Medicine, Kyorin University, 6-20-2 Shinkawa, Mitaka-shi, Tokyo 181-8611, Japan; h141@ks.kyorin-u.ac.jp; 4 Department of Pediatrics, Sapporo Medical University School of Medicine, Minami 1-jo, Nishi 16-chome, Chuo-ku, Hokkaido, Sapporo 060-8543, Japan; tsugawat@sapmed.ac.jp

In Japan, there is a proverb that the common cold is associated with all diseases. It is a simple legend, but also true in a sense. For example, seasonal influenza sometimes causes severe diseases, such as pneumonia and Reye’s syndrome [1]. The human rhinovirus may also cause not only a common cold but also virus-induced asthma [2]. In addition, the human respiratory syncytial virus (HRSV) frequently triggers asthmatic bronchiolitis in infants and the elderly [3]. Moreover, the emerging respiratory viruses, such as the new types of influenza viruses and coronaviruses, may even bring about a pandemic. Indeed, severe acute respiratory syndrome coronavirus type 2 (SARS-CoV-2) suddenly emerged in late 2019, spawning a pandemic, the coronavirus disease 2019 (COVID-19), with millions of fatalities due to respiratory failure [4]. Similarly, numerous respiratory viruses and their infections may show broader symptoms than the typical manifestations, such as nasal discharge, sneeze, cough, fever and sore throat.

Therefore, it is essential to diagnose the cause of respiratory infections accurately, treat the infections and manage the diseases effectively. We can confirm various respiratory viruses rapidly with great sensitivity and specificity using PCR methods. We can also accelerate molecular pharmacology research and drug discovery by simulating molecular interactions between anti-viral agents and viral genomes in research using advanced bioinformatics technologies [5]. Moreover, molecular epidemiology, based on advanced genomics and bioinformatics, enables us to delineate the phylogeny, genotype, antigenicity and infection route of the infectious agents [6]. Indeed, the detailed transmission routes of COVID-19 have been thus elucidated [7]. From the circumstances, we will focus on molecular epidemiology based on advanced bioinformatics technologies and survey the recent advances in diagnosing and managing various viral respiratory infections.

This Special Issue has collated many excellent articles by international researchers that present cutting-edge technologies and suggestive information. We sincerely hope these articles will help develop respiratory sciences as a new, comprehensive discipline and enable the prompt treatment of various respiratory infections.
